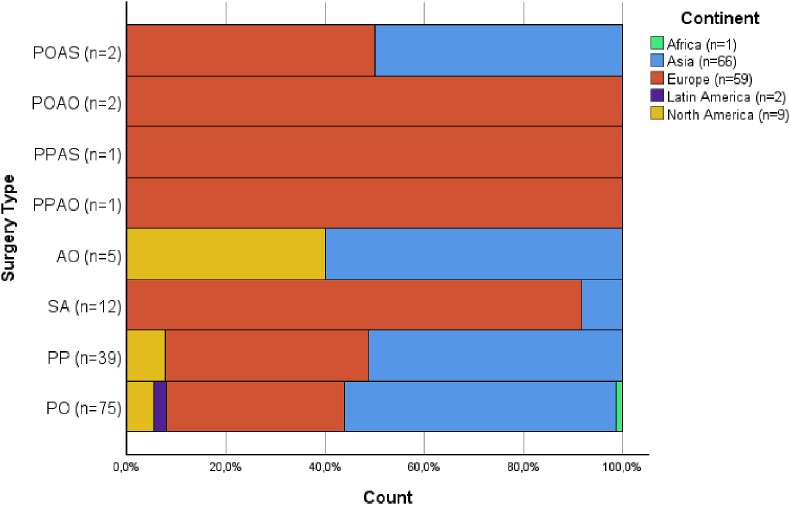# Surgical treatment of traumatic fractures of the thoracic and lumbar spine: A systematic review

**DOI:** 10.1016/j.bas.2024.102745

**Published:** 2024-01-05

**Authors:** Timon F.G. Vercoulen, Menco J.S. Niemeyer, Felix Peuker, Jorrit-Jan Verlaan, F. Cumhur Oner, Said Sadiqi

**Affiliations:** a Diakonessenhuis, Department of Orthopedic Surgery, Bosboomstraat 1, 3582, KE, Utrecht, the Netherlands; b University Medical Center Utrecht, Department of Orthopedic Surgery, Heidelberglaan 100, 3584, CX, Utrecht, the Netherlands

**Keywords:** Spine, Trauma, Thoracal, Lumbar, Fracture, Surgery

## Abstract

**Introduction:**

The treatment of traumatic thoracic and lumbar spine fractures remains controversial. To date no consensus exists on the correct choice of surgical approach and technique.

**Research question:**

to provide a comprehensive up-to-date overview of the available different surgical methods and their quantified outcomes.

**Methods:**

PubMed and EMBASE were searched between 2001 and 2020 using the term ‘spinal fractures’. Inclusion criteria were: adults, ≥10 cases, ≥12 months follow-up, thoracic or lumbar fractures, and surgery <3 weeks of trauma. Studies were categorized per surgical technique: Posterior open (PO), posterior percutaneous (PP), stand-alone vertebral body augmentation (SA), anterior scopic (AS), anterior open (AO), posterior percutaneous and anterior open (PPAO), posterior percutaneous and anterior scopic (PPAS), posterior open and anterior open (POAO) and posterior open and anterior scopic (POAS). The PO group was used as a reference group.

**Results:**

After duplicate removal 6042 articles were identified. A total of 102 articles were Included, in which 137 separate surgical technique cohorts were described: PO (n = 75), PP, (n = 39), SA (n = 12), AO (n = 5), PPAO (n = 1), PPAS (n = 1), POAO (n = 2) and POAS (n = 2).

**Discussion and conclusion:**

For type A3/A4 burst fractures, without severe neurological deficit, posterior percutaneous (PP) technique seems the safest and most feasible option in the past two decades. If needed, PP can be combined with anterior augmentation to prevent secondary kyphosis. Furthermore, posterior open (PO) technique is feasible in almost all types of fractures. Also, this technique can provide for an additional posterior decompression or fusion. Overall, no neurologic deterioration was reported following surgical intervention.

## Highlights

• Posterior percutaneous fixation seem to produce comparable results on kyphosis correction compared to the posterior open technique, with less blood loss and a shorter duration of surgery for type A3/A4 burst fractures, without severe neurological deficit.
• A posterior open technique is feasible in almost all types of fractures, and is most applied when additional posterior decompression or fusion is required, which cannot be achieved by more minimal invasive methods.
• Overall, following all surgical techniques no neurological deterioration was reported.

## 1 Introduction

Controversies remain on the optimal treatment of traumatic thoracic and lumbar spine fractures. Among different countries, regions, hospitals and even surgeons a wide range of treatment alternatives are seen, depending on their training and available materials (Vaccaro et al., 2016; Thomas et al., 2006; Abudou et al., 2013). In order to uniform the description, cluster fractures, and determine prognosis, a variety of classification systems have been introduced. Currently, the most commonly used system is the AO Spine Thoracolumbar Classification System, which incorporates radiological morphology and clinical factors (Vaccaro et al., 2013). However, as it does offer a uniform description for thoracic and lumbar fractures of the spine, it is not a treatment algorithm.

Depending on several clinical parameters, traumatic thoracic and lumbar spine fractures can be treated either surgical or conservative. Surgical treatment aims to reduce the fracture, prevent neurological deterioration, avoid further post-traumatic spinal deformity while maintaining spinal alignment and, if needed, perform a neural decompression. However, there is variety in literature regarding the optimal surgical approach and technique for different types of spine fractures. The decision depends on injury morphology, neurological status, integrity of the posterior ligamentous complex, available resources and, probably most importantly, surgeon expertise. Roughly, the types of surgical approach can be classified as anterior, posterior or combined. The choice depends on the required reduction, stabilization and neural decompression (Vaccaro et al., 2006). In 2004, Verlaan et al. published a thorough review that described the clinical relevance of the surgical treatment options for traumatic thoracic and lumbar fractures. Over the past two decades there have been various developments and less invasive techniques have become more mainstream.

Various meta-analysis and narrative reviews have been published on the treatment of traumatic thoracic and lumbar fractures of the spine (Xu et al., 2013; Lu et al., 2022; McAnany et al., 2016; Phan et al., 2015; Yi et al., 2006; Hughes et al., 2021; Sun et al., 2017; Tan et al., 2019; Kim et al., 2015; Pneumaticos et al., 2013). However, no comprehensive up-to-date overview is available of the different surgical methods, while using quantified outcome data. Therefore, the current authors have sought to provide an update on the 2004 review, using the same research team and more extensive methods (Verlaan et al., 2004).

## 2 Methods

### 2.1 Search strategy

A systematic literature search was performed in PubMed and EMBASE using the search term ‘Spinal fractures’ for articles published between 2001 and 2020. No language restrictions were imposed and a cross-reference check was performed (Appendix 1). For all missing full-texts the library of our academic institution was consulted, and the authors were e-mailed with the request to provide the full text.

### 2.2 Study selection

Article screening was performed by four researchers (TV, MN, FP, SS). Inclusion criteria were: reporting adult subjects (>18 years of age); including more than 10 cases; a minimum of 12 months of follow-up; adequate description of the surgical procedure; using generally accepted outcome instruments; surgery performed within three weeks after trauma; including only thoracic or lumbar fractures. Studies that included non-traumatic fractures of the spine were excluded. The articles meeting the inclusion and exclusion criteria were extracted for full-text analysis.

### 2.3 Data extraction

Data extraction was performed using a standardized form, simultaneously accessible for all authors (Google Spreadsheets, Google LLC, 2021/2022). Studies were categorized per surgical approach (posterior, anterior or combined) and sub-divided in one of the nine surgical technique groups.

• Posterior open (PO)
• Posterior percutaneous (PP)
• Stand-alone vertebral augmentation (SA)
• Anterior scopic (AS)
• Anterior open (AO)
• Posterior percutaneous and anterior open (PPAO)
• Posterior percutaneous and anterior scopic (PPAS)
• Posterior open and anterior open (POAO)
• Posterior open and anterior scopic (POAS).

A description of the included studies is outlined in Appendix 1.

### 2.4 Data analysis

No quality assessment could be performed. Data was analyzed using the abovementioned surgical technique groups. Descriptive statistics were applied to summarize the distribution of values per group. The PO group was considered as the reference group, since it was found to be the most applied approach and included the largest number of studies and subjects.

To calculate the Cobb angle correction loss, the difference in Cobb angle between the directly postoperative Cobb angle and the Cobb angle at follow-up were calculated for each surgical technique group. Overall correction was defined as the difference between the pre-operative Cobb angle and the Cobb angle at follow-up.

## 3 Results

**Fig. 1:**
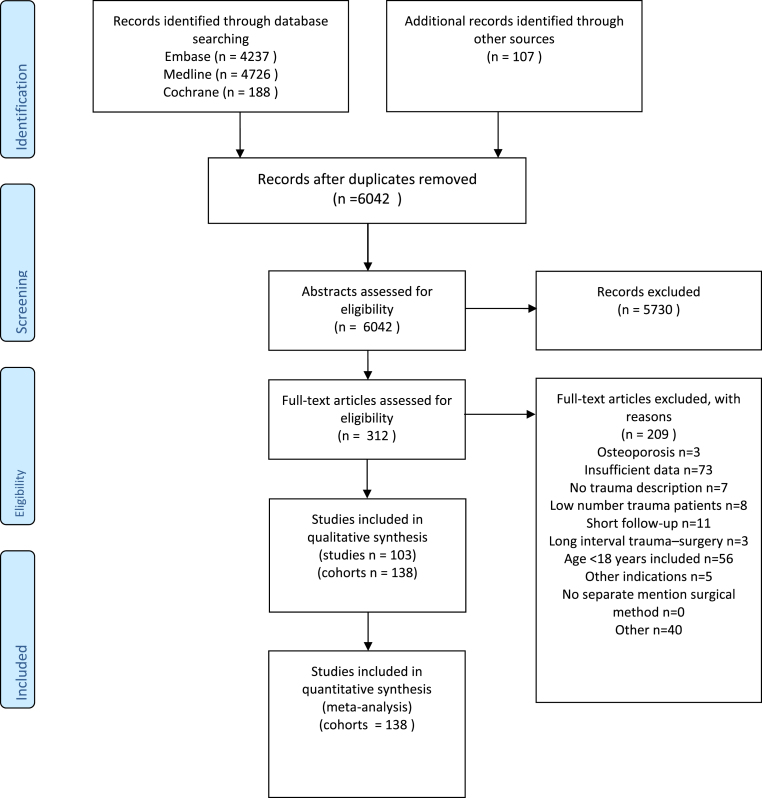
PRISMA flow chart of included studies.

In total, 9151 articles were identified following the search strategy. A cross-reference check resulted in 107 additional articles. After duplicate removal, as well as title and abstract screening, 312 articles remained for further analysis. Of these articles, 209 were excluded due to various reasons (Fig. 1). Ultimately, 102 articles were analyzed of which 137 patient cohorts could be extracted that were treated with any of the surgical techniques. Five prospective controlled trials were found comparing the following.

• different time to surgery using traditional posterior pedicle screw constructs (Cengiz et al., 2008),
• applying a traditional posterior pedicle screw construct using a less invasive posterior approach compared with an open approach (Chang et al., 2018),
• the use of a traditional posterior pedicle screw construct compared with a group where posterior fusion was added (Jindal et al., 2012),
• the use of a traditional posterior pedicle screw construct compared with a group with added laminar hooks (Korovessis et al., 2004),
• posterior fusion with instrumentation, compared with anterior fusion with instrumentation (Wood et al., 2005).

**Fig. 2:**
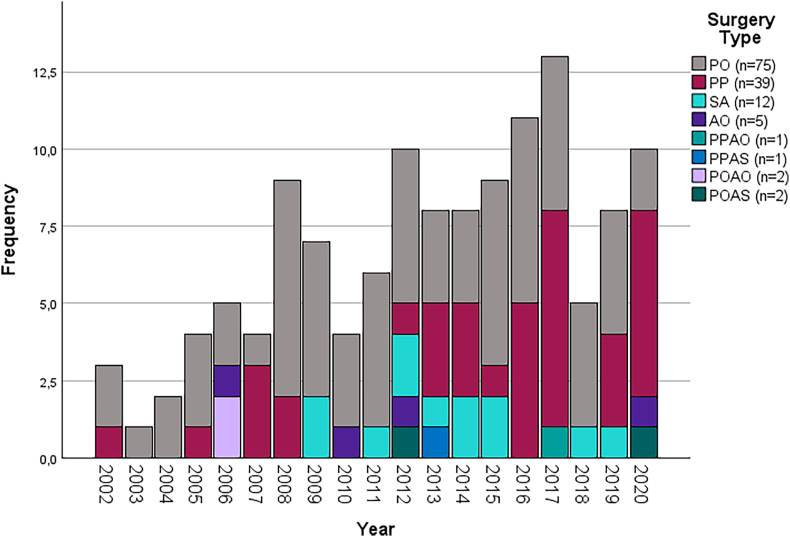
Published cohorts per year and type of surgery. *PO: Posterior open, PP: Posterior percutaneous, SA: Stand-alone vertebral body augmentation AO: Anterior open, PPAO: Posterior percutaneous and anterior open; PPAS: Posterior percutaneous and anterior scopic, POAO: Posterior open and anterior open, POAO Posterior open and anterior open, POAS: Posterior open and anterior scopic.*

The other studies were prospective or retrospective cohort studies. The posterior (PO (n = 75)17-77, PP (n = 39) (Fan et al., 2017; Kocis et al., 2020; Lee et al., 2013; Lyu et al., 2016; Pishnamaz et al., 2015; Vanek et al., 2014; Wang et al., 2017; Wang et al., 2013; Zhang et al., 2016; Zou et al., 2017; Afzal et al., 2008; Altay et al., 2007; Andress et al., 2002; Butt et al., 2007; Caruso et al., 2019; Caruso et al., 2018; Ding et al., 2021; He et al., 2013; Jiang et al., 2020; Kerschbaumer et al., 2019; Korovessis et al., 2008b; Korovessis et al., 2017; Korovessis et al., 2014; Li et al., 2016b; Manson et al., 2020; Padalkar and Mehta, 2017; Podet et al., 2020; Proietti et al., 2014; Sanli et al., 2019; Shao et al., 2020; Shin et al., 2020; Teyssedou et al., 2012; Trungu et al., 2019), SA (n = 13)) (He et al., 2013; Kerschbaumer et al., 2019; Teyssedou et al., 2012; Costa et al., 2009; Grelat et al., 2018; Hartmann et al., 2012; Hartmann et al., 2015; Klezl et al., 2011; Maestretti et al., 2014; Saget et al., 2014; Schmelzer-Schmied et al., 2009; Schulz et al., 2015), and anterior (AO (n = 5)) (Wood et al., 2005; Shin et al., 2020; Liang et al., 2017; Oskouian et al., 2006; Smith et al., 2010; Yang et al., 2012) approaches seemed to be the most often reported surgical techniques. As an anterior scopic technique only thoracoscopy was reported, in the found studies it was not used as the sole surgical technique. A combination of these (PPAO (Kreinest et al., 2017), PPAS (Spiegl et al., 2013), POAO (Korovessis et al., 2006; Payer, 2006) and POAS (Schmid et al., 2012; Tandon et al., 2020)) were studied occasionally (Fig. 2). In the past decade, there was an increase in studies including posterior percutaneous technique (PP) while a decrease in studies was observed reporting posterior open technique (PO). The largest number of articles were from Asia (n = 66) followed by Europe (n = 59) (Appendix 2). The mean year published, age, follow-up duration, gender ratio and number of polytrauma patients are reported in Table 1.

**Table 1:** General information on the study population.

Patients/cohorts	Posterior			Anterior	Combined			
	PO	PP	SA	AO	PPAO	PPAS	POAO	POAS
	2371/75	1366/39	408/12	325/5	47/1	10/1	40/2	36/2
**Year published (mean (range))**	2012	2015	2013	2012	2017	2013	2006	2016
	(2002–2020)	(2002–2020)	(2009–2019)	(2005–2020)			(2006–2006)	(2012–2020)
**follow-up (months)**	32	26	29	31	37	77	35	59,7
**range**	(12–166)	(12–72)	(12–120)	(21–45)			(24–46)	(20–99)
**mean age (years)**	41	45	50	42	47	42	38	37
**range**	(25–60)	(33–72)	(34–75)	(38–47)			(36–39)	(36–39)
**gender-ratio (M/F)**	0.66	0.62	0.55	0.64	0.55	0.60	0.75	0.67
**range**	(0.40–1.00)	(0.45–0.81)	(0.34–0.70)	(0.55–0.77)			(0.70–0.80)	(0.57–0.77)
**Polytrauma (mean, SD)**	9	25	2	NR	NR	NR	NR	NR
	(11)	(13)	(NA)					

PO: Posterior open, PP: Posterior percutaneous, SA: Stand-alone vertebral body augmentation, AO: Anterior open, PPAO: Posterior percutaneous and anterior open; PPAS: Posterior percutaneous and anterior scopic, POAO: Posterior open and anterior open, POAO Posterior open and anterior open, POAS: Posterior open and anterior scopic.

### 3.1 Pre-operative

Among the included studies, the most prevalent fracture was a burst fracture of the thoracolumbar spine (AO Spine Classification type A3 or A4). Only ten studies reported on polytrauma patient (PO (Korovessis et al., 2004; Knop et al., 2002; Leferink et al., 2003; Wang et al., 2014a; Xiong et al., 2013; Yue et al., 2002), PP (Podet et al., 2020; Sanli et al., 2019)^,^ and SA (Hartmann et al., 2012)), of which four studies included more than 50 percent of polytrauma patients (Xiong et al., 2013; Wang et al., 2014a; Yue et al., 2002; Podet et al., 2020). Most of the differences between studies were found in the pre-operative neurological status. Relatively, more serious pre-operative neurological impairment was seen in patients that received an anterior or combined (anterior and posterior) surgical approach (Table 1, Table 2).

**Table 2:** Pre-operative status of the study population.

Fracture Type (AO classification)	Posterior			Anterior	Combined			
	PO	PP	SA	AO	PPAO	PPAS	POAO	POAS
**Patients reported (n), percentage of reported (%)**	2265 (100%)	1295 (100%)	344 (100%)	217 (100%)	47 (100%)	10 (100%)	40 (100%)	36 (100%)
**A0, A1, A2**	237 (10%)	278 (21%)	209 (61%)	3 (2%)	0 (0%)	0 (0%)	0 (0%)	0 (0%)
**A3, A4**	1603 (70%)	816 (63%)	135 (39%)	154 (68%)	38 (81%)	10 (100%)	34 (85%)	36 (100%)
**B**	189 (8%)	172 (13%)	0 (0%)	47 (24%)	9 (19%)	0 (0%)	3 (8%)	0 (0%)
**C**	256 (11%)	29 (2%)	0 (0%)	13 (7%)	0 (0%)	0 (0%)	3 (8%)	0 (0%)
**Region of fracture**								
**Subjects (n), percentage of total (%)**^a^	2234 (100%)	1274 (100%)	408 (100%)	303 (100%)	47 (100%)	10 (100%)	40 (100%)	36 (100%)
**All levels**	655 (29%)	605 (47%)	252 (62%)	163 (54%)	47 (100%)	0 (0%)	0 (0%)	22 (61%)
**Thoracic**	110 (5%)	0 (0%)	0 (0%)	0 (0%)	0 (0%)	0 (0%)	0 (0%)	0 (0%)
**Thoracolumbar**	853 (38%)	478 (38%)	135 (33%)	20 (7%)	0 (0%)	10 (100%)	20 (50%)	14 (39%)
**Lumbar**	97 (4%)	16 (1%)	0 (0%)	120 (39%)	0 (0%)	0 (0%)	20 (50%)	0 (0%)
**Combination**^a^	519 (23%)	175 (14%)	21 (5%)	0 (0%)	0 (0%)	0 (0%)	0 (0%)	0 (0%)
**Neurological status (ASIA)**								
**Subjects (n), percentage of total (%)**	2318 (100%)	1266 (100%)	348 (100%)	277 (100%)	47 (100%)	10 (100%)	40 (100%)	36 (100%)
**A**	239 (10%)	21 (2%)	0 (0%)	63 (23%)	0 (0%)	0 (0%)	6 (15%)	22 (61%)
**B**	149 (6%)	38 (3%)	0 (0%)	48 (18%)	0 (0%)	0 (0%)	2 (5%)	0 (0%)
**C**	243 (11%)	64 (5%)	0 (0%)	62 (22%)	0 (0%)	0 (0%)	8 (20%)	0 (0%)
**D**	282 (12%)	62 (6%)	0 (0%)	59 (21%)	0 (0%)	0 (0%)	5 (13%)	0 (0%)
**E**	1407 (61%)	1081 (84%)	348 (100%)	45 (16%)	47 (100%)	10 (100%)	19 (48%)	14 (39%)

PO: Posterior open, PP: Posterior percutaneous, SA: Stand-alone vertebral body augmentation, AO: Anterior open, PPAO: Posterior percutaneous and anterior open; PPAS: Posterior percutaneous and anterior scopic, POAO: Posterior open and anterior open, POAO Posterior open and anterior open, POAS: Posterior open and anterior scopic.

a ‘All levels’ includes thoracic, thoracolumbar and lumbar fractures, ‘combination’ includes a combination of two of the three regions.

### 3.2 Surgical characteristics

In the PO group, transpedicular spongioplasty using mainly autologous bone grafts, or a mixture with allograft or calcium sulfate was used in 19% of the subjects. In 58% of the subjects in the PO group, an additional decompression and fusion using various types of grafts was applied. Both the PO and PP groups showed small numbers of additional vertebroplasty or kyphoplasty (3% and 1%, respectively). Less fusion, grafts, decompression, and transpedicular spongioplasty were applied in the PP group.

The mean intra-operative blood loss was 430 ml in PO and 239 ml in PP. For SA, blood loss was only reported in two studies (0 ml and 450 ml) (He et al., 2013; Verlaan et al., 2005). The reported duration of surgery was lowest in the SA group, followed by the PP group and highest in the PO group (50, 95, and 134 min, respectively) (Table 3).

**Table 3:** Surgery characteristics.

Surgery characteristics percentage of total (%)	Posterior			Anterior	Combined		
	PO	PP	SA	AO	PPAS	POAO	POAS
**Patients reported (n), percentage of reported (%)**	2312	1320	NR	NR	NR	NR	NR
**Transpedicular spongioplasty**	425 (19%)	32 (2%)	NR	NR	NR	NR	NR
**Patients reported (n)**	1548	1000	368	NR	NR	NR	NR
**Vertebro- or kyphoplasty**	45 (3%)	26 (1%)	368 (100%)	NR	NR	NR	NR
**Patients reported (n)**	1600	748	368	NR	NR	NR	NR
**Cement**	207 (13%)	52 (7%)	368 (100%)	NR	NR	NR	NR
**Patients reported (n)**	2211	1071	NR	325	10	40	36
**Decompression**	1020 (46%)	177 (17%)	NR	254 (%)	0	9 (23%)	36 (100%)
**Patients reported (n)**	2048	1118	NR	NR	10	40	36
**Fusion**	1357 (58%)	320 (29%)	NR	NR	0	40 (100%)	36 (100%)
**Graft**	1429^a^	341^b^	NR	253^c^	10^d^	40^%^	36^e^
**Patients reported (n)**	1422	774	42	274	NR	40	36
**Blood loss (ml, (SD))**	430 (332)	239 (444)	225 (317)	819 (460)	NR	1425 (107)	722 (549)
**Patients reported (n)**	1525	947	196	274	NR	40	36
**Duration (min, (SD))**	134 (58)	95 (57)	60 (44)	216 (86)	NR	288 (61)	155 (82)

PO: Posterior open, PP: Posterior percutaneous, SA: Stand-alone vertebral body augmentation, AO: Anterior open, PPAO: Posterior percutaneous and anterior open; PPAS: Posterior percutaneous and anterior scopic, POAO: Posterior open and anterior open, POAO Posterior open and anterior open, POAS: Posterior open and anterior scopic.

%: anterior: autograft cortical n = 20, both autograft cancellous and artificial; posterior: autograft cortical n = 20, allograft cancellous n = 20.

a autograft cancellous n = 271, autograft cortical n = 365, allograft cancellous n = 63, artificial n = 20, unknown n = 207, both autograft cancellous and cortical n = 130, both auto- and allograft cancellous n = 30, both autograft cancellous and artificial n = 137, both auto- and allograft cortical n = 64, allograft cancellous + cortical and autograft cortical n = 32, both auto- and allograft cancellous and cortical n = 110.

b autograft cancellous n = 50, autograft cortical n = 158, artificial n = 38, unknown n = 95.

c autograft cancellous n = 171, both autograft cancellous and cortical n = 22, autograft cancellous and allograft cortical n = 60.

d anterior: unknown n = 10; posterior: autograft cortical n = 10.

e anterior: autograft cancellous n = 14, both autograft cancellous and cortical n = 22; posterior: both autograft cancellous and cortical n = 36.

### 3.3 Post-operative

Post-operative data, including immobilization method (e.g. brace), hospital stay and complications, was reported for only 11 of the 74 cohorts. A wide variety in immobilization period was found, in mean amount of weeks this was 11 (PP), 8 (PO), 3 (SA), 1 (AO), 3 (POAO). If reported, then often a brace was used (Butt et al., 2008; Formica et al., 2016; Guo et al., 2010; Khattab and Elkhateeb, 2019; Zou et al., 2017; Butt et al., 2007; Shin et al., 2020). Moreover, the hospital stay (mean days) was 10 in three PP cohorts (Pishnamaz et al., 2015; Butt et al., 2007; Shin et al., 2020) and 7 in three PO cohorts (Formica et al., 2016; Khattab and Elkhateeb, 2019; Pishnamaz et al., 2015). Of the number of patients with reported data on post-operative complications, 4% of the PO group (Butt et al., 2008; Formica et al., 2016; Guo et al., 2010; Khattab and Elkhateeb, 2019; Pishnamaz et al., 2015; Zou et al., 2017) 10% in PP group (Pishnamaz et al., 2015; Zou et al., 2017; Butt et al., 2007) and 4% in AO group (Shin et al., 2020) had a complication of any sorts.

### 3.4 Follow-up

**Fig. 3:**
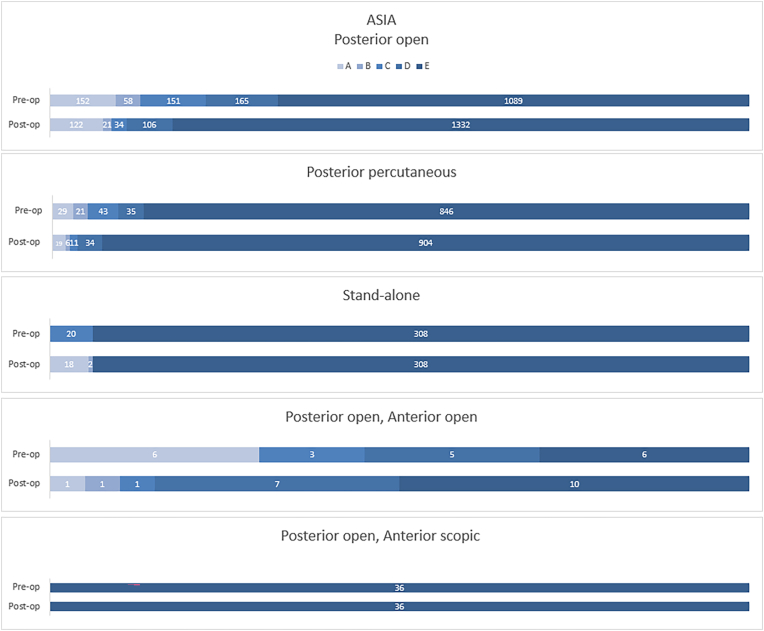
ASIA score pre-operative and post-operative per surgical method.

Reports of material failure were rare, yet occurred most following PO (2%), followed by PP (1%) and SA (0–1%). No material failure was found following AO and POAO. As shown in Table 4, reoperation rate was low in all groups, ranging from 0 to 2%. The Cobb angle pre-operatively and at follow-up was widely reported (Table 5). The lowest overall correction was found following SA (1°), the other cohorts measured a minimum of 6°. The change in ASIA score was reported in 53 cohorts (PO), 29 cohorts (PP), 10 cohorts (SA), 5 cohorts (AO), 2 cohorts (POAS) and 1 cohort (POAO). Overall, any surgical intervention led to an improvement of ASIA score or an unchanged ASIA score, with the exception of two cases of neurological deterioration. One patient in the PO group deteriorated from ASIA D to ASIA A and one in the PP group from ASIA D to ASIA B (Fig. 3) ^725^.

**Table 4:** Complications at follow-up and immobilization duration.

General follow-up data	Posterior			Anterior	Combined	
	PO	PP	SA	AO	POAO	POAS
**Patients reported (n) (cohorts reported)**	1152–1638 (36–56)	342–525 (13–20)	301–308 (10–11)	98 - 209 (2–4)	0-3 (1–2)	36 (2)
**Weeks immobilization (mean weeks), range**	8 (0–24)	10 (0–36)	3 (0–13)	2 (0–4)	3 (NA)	0
**Material failure (n, %)**	41 (3%)	7 (2%)	0	1 (0–1%)	0	NR
**Reoperation (n, %)**	27 (2%)	7 (1%)	1 (0–1%)	0	0	NR

PO: Posterior open, PP: Posterior percutaneous, SA: Stand-alone vertebral body augmentation, AO: Anterior open, POAO: Posterior open and anterior open, POAO Posterior open and anterior open, POAS: Posterior open and anterior scopic.

**Table 5:** Cobb angle pre-operative, post-operative and at follow-up. Also reported are the correction lost and overall correction at final follow-up.

	Posterior			Anterior	Combined		
	PO	PP	SA	AO	PPAO	POAO	POAS
**Pre-operative**							
**Patients reported (n)**	1944	1074	353	277	47	40	36
**Cobb (mean, SD)**	15.2 (8.1)	13.5 (6.5)	7.3 (7.7)	17.3 (2.3)	14	18.0 (2.8)	10.0 (33.9)
**Post-operative**							
**Patients reported (n)**	177	100	NR	46	NR	NR	NR
**Cobb (mean, SD)**	1.18 (2.94)	6.13 (6.53)		11.9 (NA)		NR	NR
**Follow-up**							
**Patients reported (n)**	1762	938	344	277	NR	40	14
**Follow-up duration (range)**	34 (12–166)*	25 (12–72)	34 (12–120)	29 (21–45)		35 (24–46)	20
**Cobb (mean, SD)**	7.65 (5.99)	7.23 (4.65)	6.09 (9.29)	9.68 (7.98)		7.65 (9.40)	−2.40
**Relations**							
**Patients reported (n)**	177	100	NR	46	NR	NR	NR
**Correction lost (mean, SD)**	6.42 (2.25)	2.10 (5.56)		9.10 (NA)		NR	NR
**Patients reported (n), (cohorts reported)**	1706 (56)	962 (29)	323 (8)	277 (4)	NR	40 (2)	14 (1)
**Overall correction (mean, SD)**	7.50 (6.70)	6.39 (5.23)	1.06 (4.31)	7.65 (7.91)		10.35 (6.58)	11.6 (NA)

PO: Posterior open, PP: Posterior percutaneous, SA: Stand-alone vertebral body augmentation, AO: Anterior open, PPAO: Posterior percutaneous and anterior open; PPAS: Posterior percutaneous and anterior scopic, POAO: Posterior open and anterior open, POAO Posterior open and anterior open, POAS: Posterior open and anterior scopic; SD: Standard deviation; NR: Not reported; NA: Not applicable.

## 4 Discussion

As this study shows, there has been a substantial number of publications on the surgical treatment of traumatic thoracic and lumbar spine fractures. However, the quality of the available evidence is low, with only a small number of randomized controlled trials (RCT). Various surgical treatment techniques have been compared in mostly mono-center retrospective cohort studies, with incomparable results due to a difference in follow-up time points and outcomes. This comprehensive systematic review quantifies the different outcomes data of all available studies concerning surgical treatment of traumatic thoracic and lumbar spine fractures over the past two decades. The quantity of the available data provides the best possible means to evaluate trends in surgical methods.

It is notable that the posterior percutaneous technique (PP) has been gaining in popularity in recent years. The posterior percutaneous technique is applied in similar rates and spinal regions in patients with type A3/A4 burst fractures and type B distraction injury fractures. It also measures comparable results to the posterior open technique (PO) regarding kyphosis correction, with less blood loss and a shorter surgery duration. Overall, the postoperative immobilization (e.g., with a brace) period seemed slightly less compared to PO the hospital length of stay in the PP group was relatively longer. This could be explained due to the low amount of studies that reported on this subject and the differences between the patient population of the studies. The included studies by Phan et al. (2015) and Lu et al. (2022) compared PP with PO and reported a shorter hospital stay, shorter operation time, lower infection rates and better pain scores following surgery, while maintaining similar radiographic outcome data (Lu et al., 2022; Phan et al., 2015). However, PP requires a longer learning curve and may expose the surgeon to more radiation (Court and Vincent, 2012). Moreover, it is rarely used in combination with neural decompression (Wood et al., 2005; Fan et al., 2017; Kocis et al., 2020; Lee et al., 2013; Lyu et al., 2016; Pishnamaz et al., 2015; Vanek et al., 2014; Wang et al., 2017; Wang et al., 2013; Zhang et al., 2016; Zou et al., 2017; Afzal et al., 2008; Altay et al., 2007; Andress et al., 2002; Butt et al., 2007; Caruso et al., 2019; Caruso et al., 2018; Ding et al., 2021; He et al., 2013; Jiang et al., 2020; Kerschbaumer et al., 2019; Korovessis et al., 2008b; Korovessis et al., 2017; Korovessis et al., 2014; Li et al., 2016b; Manson et al., 2020; Padalkar and Mehta, 2017; Podet et al., 2020; Proietti et al., 2014; Sanli et al., 2019; Shao et al., 2020; Shin et al., 2020; Teyssedou et al., 2012; Trungu et al., 2019).

Between 2009 and 2015 there was a rise in stand-alone vertebral augmentation (SA) use without fixation, mainly in Europe. Furthermore, only data of neurological intact patients that sustained a type A fracture was available. Advantages of the SA procedures include its minimal invasive approach, restoration of kyphosis, maintaining kyphosis correction and early pain reduction (He et al., 2013; Kerschbaumer et al., 2019; Teyssedou et al., 2012; Costa et al., 2009; Grelat et al., 2018; Hartmann et al., 2012; Hartmann et al., 2015; Klezl et al., 2011; Maestretti et al., 2014; Saget et al., 2014; Schmelzer-Schmied et al., 2009; Schulz et al., 2015; Verlaan et al., 2005). This procedure is mainly compared to conservative treatment with a brace, or a combination of posterior fixation and vertebroplasty or kyphoplasty. Three studies in the current review compared PP combined with SA to SA alone, and found that a combination of SA and PP had more favorable results (He et al., 2013; Kerschbaumer et al., 2019). This may be indicated when there is an A3 or A4 fracture with significant comminution or severely affected endplates. In this situation, protrusion of the intervertebral disc can occur through the endplates, possibly leading to failure of kyphosis correction (Teyssedou et al., 2012; Saget et al., 2014; Verlaan et al., 2005). The result of SA and PP combined was most favorable in a study by He et al. (2013), where only patients older than 65 years were included (He et al., 2013). This could be because of the increased benefit for elderly patients from anterior reinforcement and additional posterior reinforcement of the spine. The beneficial effect might be explained due to pre-existent sagittal- and coronal balance problems, possibly causing higher acting forces on the implants (Benoist, 2003). Furthermore, It should be noted that the risk of (osteoporotic) fractures may be higher in these patients, possibly resulting in different fracture characteristics limiting comparison to traumatic fractures (Waterloo et al., 2012). Although studies with osteoporotic fractures were excluded, age-related loss of bone density may have affected these results. The Deutsche Gesellschaft für Orthopädie und Unfallchirurgie (DGOU) Osteoporotic Fracture Classification System makes a clear distinction in fracture assessment for osteoporotic fractures of the spine (Schnake et al., 2018). This distinction should be acknowledged in future studies, and different treatment strategies may be needed separately for non-osteoporotic vertebral fractures and osteoporotic vertebral fractures.

The studies on anterior approaches only addressed the open variant. A relatively large number of patients with neurological deficit and more severe fractures were included. One of the studies used a procedure called selective corpectomy, in which the vertebral body was spared as much as possible to reduce the risk of cage subsidence (Liang et al., 2017). Advantages mentioned in this study include a more thorough neural decompression and clearance of the spinal canal, the need of stabilization of fewer segments for the same strength of the construct compared to posterior fusion (Allain, 2011; Xu et al., 2013). However, it should be noted that no difference in neurological recovery was found. This is also found in the study by Xu et al., in which the open anterior (AO) approach was associated with longer operative time, similar neurological outcome, greater blood loss and higher costs than the posterior open (PO) approach for thoracolumbar fractures (Xu et al., 2013). Despite only few studies reported on complication rate, similar rate of complications were reported between both procedures (Shin et al., 2020; Liang et al., 2017; Oskouian et al., 2006; Smith et al., 2010; Yang et al., 2012). It is likely that the complication and immobilization rates is underreported in literature.

### 4.1 Implications

Due to the variability in study design of the included articles, these findings should be interpreted with care. The authors would like to emphasize the importance of a uniform method to report the outcome of after treatment of traumatic thoracic and lumbar spine fractures. To standardize reporting, it is important to classify fractures according to the common and widely used classification systems, document neurological recovery according to a uniform system and report on relevant surgical characteristics (e.g., complication rate). Furthermore, the authors of this study would like to acknowledge the importance of including patient-reported outcomes for evaluating the success of surgical techniques during follow-up moment, besides using merely radiographic angles. Recently, the AO Spine PROST (Patient Reported Outcome Spine Trauma) was developed as the first patient-reported outcome measure specifically designed for spine trauma patients (Sadiqi et al., 2021; Sadiqi et al., 2017; Sadiqi et al., 2020).

### 4.2 Limitations

This systematic review has several limitations. There is a high probability of selection bias and publication bias, due to the retrospective nature and low sample sizes in most studies. However, due to the vast quantity of included studies, this study exemplifies a contemporary trend of the current practice of the different surgical techniques. Unfortunately, no meta-analysis could be performed due to the heterogeneity in methodology, follow-up, and outcome measures used in the included studies. Moreover, various important injury and patient characteristics—e.g., the frequency and severity of polytrauma injuries—were rarely reported.

## 5 Conclusion

In conclusion, this comprehensive systematic review describes and compares quantitative data regarding different surgical techniques for the treatment of traumatic thoracic and lumbar spine fractures. For type A3/A4 burst fractures without severe neurological deficit, posterior percutaneous (PP) technique seems the safest and most feasible option. If needed, PP can be combined with anterior augmentation in order to prevent secondary kyphosis in communitive fractures or severely affected endplates. Furthermore, posterior open (PO) technique is feasible in almost all types of fractures. Also, there seems to be particular use for this technique when additional posterior decompression or fusion is required, which cannot be achieved by more minimal invasive methods. Overall, no neurologic deterioration was reported following surgical intervention. Randomized controlled trials are imperative for a true comparison of surgical methods, and unfortunately were scarcely available. This systematic review exhibits a comprehensive overview of trends and clinical outcomes related to most common surgical treatment strategies in literature for patients with traumatic thoracic and lumbar spine fractures.

## Declaration of competing interest

The authors declare the following financial interests/personal relationships which may be considered as potential competing interests: J.J. Verlaan reports a relationship with 10.13039/100004320Philips Healthcare that includes: funding grants. J.J. Verlaan reports a relationship with SentryX that includes: board membership and equity or stocks. F.C. Oner reports a relationship with AO spine knowledge forum trauma that includes: board membership. F.C. Öner guest editor Brain and Spine If there are other authors, they declare that they have no known competing financial interests or personal relationships that could have appeared to influence the work reported in this paper.

## Figures and Tables

**Table:** 

**Author**	**Year**	**Country**	**Continent**	**Surgical method**	**Study design**	**Subjects (n)**	**Follow-up (mos)**
Amelot et al. (½) (Amelot et al., 2017)	2017	France	Europe	Posterior open	Retrospective	25	13
Amelot et al. (½) (Amelot et al., 2017)	2017	France	Europe	Posterior open	Retrospective	82	19
Aono et al. (Aono et al., 2016)	2016	Japan	Asia	Posterior open	Retro-/prospective	27	50
Butt et al. (Butt et al., 2008)	2008	India	Asia	Posterior open	Prospective	120	25
Cengiz et al. (½) (Cengiz et al., 2008)	2008	Turkey	Europe	Posterior open	RCT	12	15
Cengiz et al. (½) (Cengiz et al., 2008)	2008	Turkey	Europe	Posterior open	RCT	15	15
Chang et al. (½) (Chang et al., 2018)	2018	China	Asia	Posterior open	RCT	30	28
Chang et al. (½) (Chang et al., 2018)	2018	China	Asia	Posterior open	RCT	32	29
Chokshi et al. (Chokshi and Shah, 2019)	2019	India	Asia	Posterior open	Prospective	50	18
Dai et al. (½) (Dai et al., 2009)	2009	China	Asia	Posterior open	Prospective	37	72
Dai et al. (½) (Dai et al., 2009)	2009	China	Asia	Posterior open	Prospective	36	72
De Gendt et al. (De Gendt et al., 2020)	2020	Netherlands	Europe	Posterior open	Prospective	20	166
Defino et al. (Defino and Canto, 2007)	2007	Brazil	Latin America	Posterior open	Prospective	20	24
Dobran et al. (½) (Dobran et al., 2016)	2016	Italy	Europe	Posterior open	Retrospective	30	33
Dobran et al. (½) (Dobran et al., 2016)	2016	Italy	Europe	Posterior open	Retrospective	30	31
Fan et al. (½) (Fan et al., 2017)	2017	China	Asia	Posterior open	Retrospective	49	16
Farrokhi et al. (½) (Farrokhi et al., 2010)	2010	Iran	Asia	Posterior open	Prospective	42	37
Farrokhi et al. (½) (Farrokhi et al., 2010)	2010	Iran	Asia	Posterior open	Prospective	38	37
Fisher et al. (Fisher et al., 2009)	2009	Canada	North America	Posterior open	Retro-/prospective	27	38
Formica et al. (Formica et al., 2016)	2016	Italy	Europe	Posterior open	Retrospective	43	12
Ge et al. (Ge et al., 2011)	2011	China	Asia	Posterior open	Prospective	41	33
Guo et al. (Guo et al., 2010)	2010	China	Asia	Posterior open	Prospective	42	15
Hadgaonkar et al. (Hadgaonkar et al., 2017)	2017	India	Asia	Posterior open	Prospective	11	24
Hariri et al. (Hariri et al., 2018)	2018	USA	North America	Posterior open	Retrospective	64	12
Hofstetter et al. (Hofstetter et al., 2011)	2011	USA	North America	Posterior open	Retrospective	17	21
Islam et al. (Islam et al., 2011)	2011	Dhaka	Asia	Posterior open	Prospective	16	14
Jindal et al. (½) (Jindal et al., 2012)	2012	India	Asia	Posterior open	RCT	23	24
Jindal et al. (½) (Jindal et al., 2012)	2012	India	Asia	Posterior open	RCT	24	24
Khattab et al. (Khattab and Elkhateeb, 2019)	2019	Egypt	Africa	Posterior open	Prospective	27	38
Knop et al. (Knop et al., 2002)	2002	Germany	Europe	Posterior open	Retrospective	29	42
Kocis et al. (½) (Kocis et al., 2020)	2020	Czech Republic	Europe	Posterior open	Prospective	23	12
Korovessis et al. (½) (Korovessis et al., 2004)	2004	Greece	Europe	Posterior open	RCT	20	52
Korovessis et al. (Korovessis et al., 2008a)	2008	Greece	Europe	Posterior open	Prospective	23	29
Korovessis et al. (½) (Korovessis et al., 2004)	2004	Greece	Europe	Posterior open	RCT	20	52
Korovessis et al. (½) (Korovessis et al., 2006)	2006	Greece	Europe	Posterior open	Prospective	20	48
Lee et al. (½) (Lee et al., 2013)	2013	South Korea	Asia	Posterior open	Retrospective	27	40
Leferink et al. (Leferink et al., 2003)	2003	Netherlands	Europe	Posterior open	Retrospective	19	54
Li et al. (½) (Li et al., 2012)	2012	China	Asia	Posterior open	Retrospective	30	13
Li et al. (½) (Li et al., 2005)	2005	China	Asia	Posterior open	Retrospective	75	37
Li et al. (Li et al., 2016a)	2016	China	Asia	Posterior open	Retrospective	50	12
Li et al. (½) (Li et al., 2005)	2005	China	Asia	Posterior open	Retrospective	45	40
Li et al. (½) (Li et al., 2012)	2012	China	Asia	Posterior open	Retrospective	30	13
Liu et al. (½) (Liu et al., 2015)	2015	China	Asia	Posterior open	Retrospective	33	53
Liu et al. (½) (Liu et al., 2015)	2015	China	Asia	Posterior open	Retrospective	30	48
Lyu et al. (⅓) (Lyu et al., 2016)	2016	China	Asia	Posterior open	Prospective	30	18
Maior et al. (Maior et al., 2018)	2018	Romania	Europe	Posterior open	Retrospective	44	12
Marré et al. (Marre et al., 2011)	2011	Chili	Latin America	Posterior open	Retrospective	51	89
Masuda et al. (Masuda et al., 2015)	2015	Japan	Asia	Posterior open	Retrospective	11	50
Modi et al. (Modi et al., 2009)	2009	South Korea	Asia	Posterior open	Retrospective	31	34
Ökten et al. (½) (Okten et al., 2015)	2015	Turkey	Europe	Posterior open	Retrospective	35	27
Ökten et al. (½) (Okten et al., 2015)	2015	Turkey	Europe	Posterior open	Retrospective	35	27
Pishnamaz et al. (½) (Pishnamaz et al., 2015)	2015	Germany	Europe	Posterior open	Prospective	43	43
Schmid et al. (½) (Schmid et al., 2012)	2012	Austria	Europe	Posterior open	Prospective	21	20
Scholz et al. (Scholz et al., 2018)	2018	Germany	Europe	Posterior open	Prospective	10	24
Siebenga et al. (Siebenga et al., 2006)	2006	Netherlands	Europe	Posterior open	Prospective	17	52
Van Herck et al. (Van Herck et al., 2009)	2009	Belgium	Europe	Posterior open	Retrospective	30	24
Vanek et al. (½) (Vanek et al., 2014)	2014	Czech Republic	Europe	Posterior open	Prospective	17	14
Verlaan et al. (Verlaan et al., 2005)	2005	The Netherlands	Europe	Posterior open	Prospective		
Verlaan et al. (Verlaan et al., 2015)	2015	Netherlands	Europe	Posterior open	Prospective	20	72
Wang et al. (½) (Wang et al., 2008a)	2008	China	Asia	Posterior open	Retrospective	20	Unknown
Wang et al. (Wang et al., 2008b)	2008	China	Asia	Posterior open	Retrospective	27	32
Wang et al. (Wang et al., 2014b)	2014	China	Asia	Posterior open	Retrospective	30	38
Wang et al.^127^	2013	China	Asia	Posterior open	Retrospective	10	22
Wang et al. (Wang et al., 2014a)	2014	China	Asia	Posterior open	Prospective	20	30
Wang et al. (½) (Wang et al., 2008a)	2008	China	Asia	Posterior open	Retrospective	50	Unknown
Wang et al. (½) (Wang et al., 2017)	2017	China	Asia	Posterior open	Retrospective	49	23
Wu et al. (Wu et al., 2016)	2016	China	Asia	Posterior open	Prospective	62	28
Xiong et al. (Xiong et al., 2013)	2013	China	Asia	Posterior open	Retrospective	11	21
Yaldiz et al. (Yaldiz et al., 2015)	2015	Turkey	Europe	Posterior open	Retrospective	20	12
Yue et al. (Yue et al., 2002)	2002	USA	North America	Posterior open	Prospective	32	22
Yung et al. (Yung and Thng, 2011)	2011	Singapore	Asia	Posterior open	Retrospective	19	18
Zhang et al. (½) (Zhang et al., 2019)	2019	China	Asia	Posterior open	Retrospective	21	12
Zhang et al. (½) (Zhang et al., 2019)	2019	China	Asia	Posterior open	Retrospective	29	12
Zhang et al. (½) (Zhang et al., 2016)	2016	China	Asia	Posterior open	Prospective	30	12
Zou et al. (½) (Zou et al., 2017)	2017	China	Asia	Posterior open	Retrospective	22	15
Afzal et al. (Afzal et al., 2008)	2008	India	Asia	Posterior percutaneous	Retrospective	16	22
Altay et al. (½) (Altay et al., 2007)	2007	Turkey	Asia	Posterior percutaneous	Retrospective	31	33
Altay et al. (½) (Altay et al., 2007)	2007	Turkey	Asia	Posterior percutaneous	Retrospective	32	36
Andress et al. (Andress et al., 2002)	2002	Germany	Europe	Posterior percutaneous	Retrospective	50	68
Butt et al. (Butt et al., 2007)	2007	India	Asia	Posterior percutaneous	Prospective	50	59
Caruso et al. (Caruso et al., 2019)	2019	Italy	Europe	Posterior percutaneous	Retrospective	32	41
Caruso et al. (½) (Caruso et al., 2018)	2018	Italy	Europe	Posterior percutaneous	Retrospective	23	12
Caruso et al. (½) (Caruso et al., 2018)	2018	Italy	Europe	Posterior percutaneous	Retrospective	26	12
Ding et al. (Ding et al., 2021)	2021	China	Asia	Posterior percutaneous	Prospective	24	19
Fan et al. (½) (Fan et al., 2017)	2017	China	Asia	Posterior percutaneous	Retrospective	63	16
He et al. (½) (He et al., 2013)	2013	China	Asia	Posterior percutaneous	Prospective	21	34
Jiang et al. (½) (Jiang et al., 2020)	2020	China	Asia	Posterior percutaneous	Retrospective	56	12
Jiang et al. (½) (Jiang et al., 2020)	2020	China	Asia	Posterior percutaneous	Retrospective	60	12
Kerschbaumer et al. (½) (Kerschbaumer et al., 2019)	2019	France	Europe	Posterior percutaneous	Prospective	14	28
Kocis et al. (½) (Kocis et al., 2020)	2020	Czech Republic	Europe	Posterior percutaneous	Prospective	23	12
Korovessis et al. (Korovessis et al., 2017)	2017	Greece	Europe	Posterior percutaneous	Retrospective	36	31
Korovessis et al. (Korovessis et al., 2014)	2014	Greece	Europe	Posterior percutaneous	Prospective	20	17
Korovessis et al. (Korovessis et al., 2008b)	2008	Greece	Europe	Posterior percutaneous	Prospective	18	22
Lee et al. (½) (Lee et al., 2013)	2013	South Korea	Asia	Posterior percutaneous	Retrospective	32	30
Li et al. (½) (Li et al., 2016b)	2016	China	Asia	Posterior percutaneous	Retrospective	16	12
Li et al. (½) (Li et al., 2016b)	2016	China	Asia	Posterior percutaneous	Retrospective	16	12
Lyu et al. (⅓) (Lyu et al., 2016)	2016	China	Asia	Posterior percutaneous	Prospective	30	18
Lyu et al. (⅓) (Lyu et al., 2016)	2016	China	Asia	Posterior percutaneous	Prospective	30	18
Manson et al. (Manson et al., 2020)	2020	Canada	North America	Posterior percutaneous	Prospective	32	24
Padalkar et al. (Padalkar and Mehta, 2017)	2017	India	Asia	Posterior percutaneous	Retrospective	25	22
Pishnamaz et al. (½) (Pishnamaz et al., 2015)	2015	Germany	Europe	Posterior percutaneous	Prospective	29	29
Podet et al. (Podet et al., 2020)	2020	USA	North America	Posterior percutaneous	Retrospective	59	14
Proietti et al. (Proietti et al., 2014)	2014	Italy	Europe	Posterior percutaneous	Retrospective	63	12
Sanli et al. (Sanli et al., 2019)	2019	Netherlands	Europe	Posterior percutaneous	Prospective	69	19
Shao et al. (Shao et al., 2020)	2020	China	Asia	Posterior percutaneous	Retrospective	22	36
Shin et al. (½) (Shin et al., 2020)	2020	South Korea	Asia	Posterior percutaneous	Retrospective	24	15
Teyssedou et al. (½) (Teyssedou et al., 2012)	2012	France	Europe	Posterior percutaneous	Prospective	16	12
Trungu et al. (½) (Trungu et al., 2019)	2019	Italy	Europe	Posterior percutaneous	Retrospective	73	72
Trungu et al. (½) (Trungu et al., 2019)	2019	Italy	Europe	Posterior percutaneous	Retrospective	71	72
Vanek et al. (½) (Vanek et al., 2014)	2014	Czech Republic	Europe	Posterior percutaneous	Prospective	18	24
Wang et al. (Wang et al., 2013)	2013	China	Asia	Posterior percutaneous	Retrospective	26	24
Wang et al. (½) (Wang et al., 2017)	2017	China	Asia	Posterior percutaneous	Retrospective	56	23
Wood et al. (Wood et al., 2005)	2005	USA	North America	Posterior percutaneous	RCT	38	43
Zhang et al. (½) (Zhang et al., 2016)	2016	China	Asia	Posterior percutaneous	Prospective	29	12
Zou et al. (½) (Zou et al., 2017)	2017	China	Asia	Posterior percutaneous	Retrospective	21	15
Costa et al. (Costa et al., 2009)	2009	Italy	Europe	Stand-alone cement augmentation	Prospective	34	12
Grelat et al. (Grelat et al., 2018)	2018	France	Europe	Stand-alone cement augmentation	Retrospective	77	44
Hartmann et al. (Hartmann et al., 2012)	2012	Germany	Europe	Stand-alone cement augmentation	Retrospective	26	15
Hartmann et al. (Hartmann et al., 2015)	2015	Germany	Europe	Stand-alone cement augmentation	Retrospective	18	27
He et al. (½) (He et al., 2013)	2013	China	Asia	Stand-alone cement augmentation	Prospective	22	34
Kerschbaumer et al. (½) (Kerschbaumer et al., 2019)	2019	France	Europe	Stand-alone cement augmentation	Prospective	60	28
Klezl et al. (Klezl et al., 2011)	2011	UK	Europe	Stand-alone cement augmentation	Prospective	10	12
Maestretti et al. (Maestretti et al., 2014)	2014	Switzerland	Europe	Stand-alone cement augmentation	Prospective	21	120
Saget et al. (Saget et al., 2014)	2014	France	Europe	Stand-alone cement augmentation	Prospective	54	24
Schmelzer-Schmied et al. (Schmelzer-Schmied et al., 2009)	2009	Germany	Europe	Stand-alone cement augmentation	Prospective	20	12
Schulz et al. (Schulz et al., 2015)	2015	Germany	Europe	Stand-alone cement augmentation	Retrospective	17	12
Teyssedou et al. (½) (Teyssedou et al., 2012)	2012	France	Europe	Stand-alone cement augmentation	Prospective	49	12
Liang et al. (Liang et al., 2017)	2017	China	Asia	Anterior open	Unknown	120	24
Oskouian et al. (Oskouian et al., 2006)	2006	USA	North America	Anterior open	Retrospective	60	21
Shin et al. (½) (Shin et al., 2020)	2020	South Korea	Asia	Anterior open	Retrospective	46	45
Smith et al. (Smith et al., 2010)	2010	USA	North America	Anterior open	Retro-/prospective	52	24
Wood et al. (Wood et al., 2005)	2005	USA	North America	Anterior open			
Yang et al. (Yang et al., 2012)	2012	China	Asia	Anterior open	Retrospective	51	26
Kreinest et al. (Kreinest et al., 2017)	2017	Germany	Europe	Posterior percutaneous, anterior open	Prospective	47	37
Spiegl et al. (Spiegl et al., 2013)	2013	Germany	Europe	Posterior percutaneous, anterior scopic	Retrospective	10	77
Korovessis et al. (½) (Korovessis et al., 2006)	2006	Greece	Europe	Posterior open, anterior open	Prospective	20	46
Payer et al. (Payer, 2006)	2006	Switzerland	Europe	Posterior open, anterior open	Prospective	20	24
Schmid et al. (½) (Schmid et al., 2012)	2012	Austria	Europe	Posterior open, anterior scopic	Prospective	14	20
Tandon et al. (Tandon et al., 2020)	2020	India	Asia	Posterior open, anterior scopic	Retrospective	22	99

**Figure:**